# Seismic anisotropy to investigate lithospheric-scale tectonic structures and mantle dynamics in southern Italy

**DOI:** 10.1038/s41598-023-47973-1

**Published:** 2023-11-28

**Authors:** L. Scarfì, M. Firetto Carlino, C. Musumeci

**Affiliations:** Istituto Nazionale di Geofisica e Vulcanologia – Osservatorio Etneo, Catania, Italy

**Keywords:** Geodynamics, Seismology, Tectonics

## Abstract

Subduction zones may be characterised by deep-seated tectonic structures whose effects propagate to the upper plate through faulting and magmatism. The overall geodynamic framework, as well as the roots of the many active faults affecting such regions, can be investigated by the study of the upper mantle anisotropic patterns, through the analysis of core-transiting teleseismic phases. Here, we discuss the results of XKS waves splitting observed in the central Mediterranean, particularly in southern Italy, which is characterised by the Adriatic-Ionian subduction system. Azimuths of polarisation of the fast wave (fast directions) were found to be generally trench-parallel, as an effect of the subducting slab, albeit a change to a perpendicular direction, in central Italy and Sicily, suggests discontinuities in the structure of the slab itself. However, while in central Italy a gradual rotation of fast directions points to a toroidal upper mantle flow through a tear in the Apenninic slab, in central-eastern Sicily, the splitting parameters show an abrupt change that matches well with the main crustal tectonic structures. There, the rapid trench migration, taking place at the transition between the subduction and continental collision domains, produced a rather complex Subduction Transform Edge Propagator fault system. The sharp variation in the pattern of the upper mantle anisotropy marks the main element of such a fault system and suggests its primary role in the segmentation process of the collisional margin. Our findings further show that the study of seismic anisotropy may be fundamental in investigating whether tectonic structures only involve the crust or extend down to the upper mantle.

## Introduction

**Figure 1:**
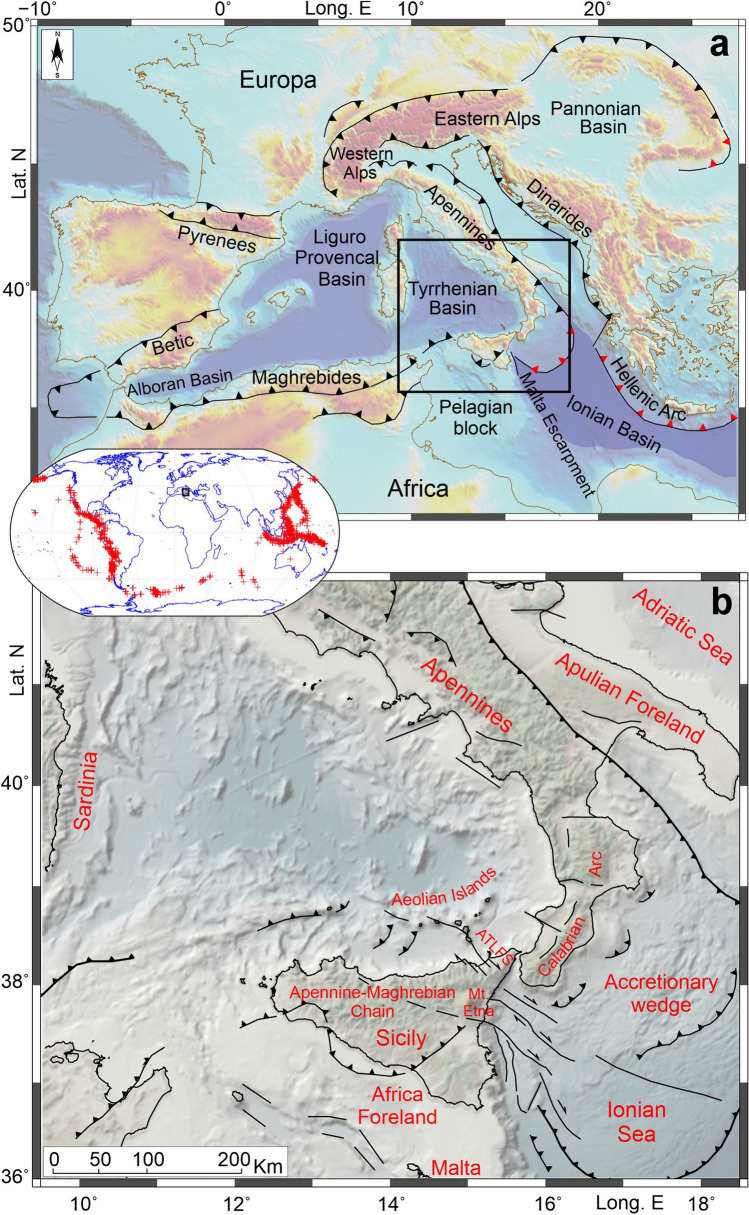
(**a**) Tectonic sketch of the Mediterranean showing the main orogenic belts, basins and subduction zones. The sawteeth points in the direction of subduction or underthrusting (in red where the subducting slab is considered to be continuous). The black box shows the area investigated in the present study. (**b**) Main tectonic features of the studied region. ATLFS, Aeolian-Tindari-Letojanni Fault System. The central inset shows the distribution of analysed earthquakes. Topography is from Ref.^67^. The maps were created using Global Mapper (version 24.0.2; https://globalmapper.it/), Generic Mapping Tools (version 6.0; https://www.generic-mapping-tools.org/) and MATLAB (version R2023a; https://it.mathworks.com/products/matlab.html).

The central Mediterranean is a geodynamically very complex area; its present-day structure is the result of a long-lasting geotectonic evolution, which, in the context of the Nubia and Eurasian plates convergence, in the last 30–35 Ma has been characterised by the opening of large-scale back-arc extensional basins (i.e. Liguro-Provençal, Algerian, Alboran, and Tyrrhenian basins), caused by the rapid south-eastward rolling back of the Ionian slab^1^ (Fig. 1). These processes have resulted in a relative rotation and arcuate shaping of the Apennine-Maghrebian Chain, a large orogenic domain extending from the Maghrebian region in northeastern Africa to the Apennines in Italy, across the Calabrian Arc^2^. Slab rollback was also accompanied by detachments and lateral tearing of the subducting lithosphere, so that the active portion of the subduction system has gradually become narrower and is today confined between central Calabria and northeastern Sicily (see Ref.^3^ and references therein). In particular, the current southern edge of the still active (and continuous at depth) slab portion is hypothesised to be located in correspondence with a fault system that extends from the central sector of the Aeolian Islands as far as the Ionian offshore, passing through the northeastern Sicilian mainland (Aeolian-Tindari-Letojanni Fault System, ATLFS in Fig. 1). West of ATLFS, a slab fragment, which is completely detached at shallow levels, has been detected in seismic tomography images (e.g. Ref.^3^).

While collisional-related compressive tectonics prevails in the southwestern margin of the Tyrrhenian Sea and in central and western Sicily^4,5^, northeastern Sicily, the Calabrian Arc and southern Apennines are mainly affected by extensional and transcurrent tectonics superimposed on the previous collisional context^6–11^. Several theories have been proposed to explain such extensional and strike-slip dynamics affecting these areas, such as an isostatic response to the progressive Ionian slab subduction^6,12^, an elastic rebound^13^ by the slab detachment^14^, an asthenospheric flow pushing on the retreating slab^15^ or even an eastward dragging of the Ionian-Calabrian domain by the Hellenic slab pull^16^. The lack of convergence towards a unique model testifies that the geodynamics of this pivotal area of the Mediterranean is far from being satisfactorily understood.

Relevant constraints on the upper Earth’s mantle layout can be derived by studying its anisotropic structure^17,18^. Indeed, such a condition yields the splitting of S-phases into two separate shear waves, whose polarisation and the relative difference in arrival times may unravel deformational processes involving the asthenosphere and lithosphere.

Taking advantage of the dense seismic coverage that has become available for the entire Italian peninsula in the last two decades, here we present splitting measurements on a larger-than-ever dataset of core-transiting seismic shear waves (i.e. PKS, SKS, SKKS phases, and others, all called XKS phases) regarding southern Italy; further, the results are analysed to relate mantle dynamic processes to surface structures in the collisional context of the central Mediterranean.

## Splitting of XKS phases

Although directionality in the physical properties of rock volumes always subsists at different scale and strength, isotropy is a good first approximation for describing seismic waves travelling through the Earth. However, markedly anisotropic materials may cause seismic energy to split into two perpendicularly polarised waves that travel at different velocities; this phenomenon, particularly evident for S waves and termed ‘shear wave splitting’, is the seismological analogue to optical birefringence^17,18^. The ‘splitting parameters’, ɸ (azimuth of polarisation of the fast wave, or else named ‘fast direction’) and dt (delay time between the fast and the slow waves first arrivals), depend on the properties of the anisotropic medium and the path length, and can be used to investigate the Earth’s interior. In particular, within the mantle, anisotropy is thought to be mainly related to the strain-induced preferred orientation of mineral grains (lattice-preferred orientation, LPO^17–19^).

In order to investigate the relationships between surface processes and mantle dynamics (the case in this study), core-transiting XKS waves from teleseismic earthquakes can be successfully used. For those earthquakes occurring at epicentral distances comprised between about 85° and 140°, XKS waves may be registered; such phases are well-separated from others and are characterised by near-vertical incidence angles, which makes them relatively easy to analyse, and avoids any amplitude and phase distortion^17,20^. They derive from P waves travelling within the liquid outer core, then converted to S waves as they pass back into the mantle. There, their particle motion is entirely along the radial direction, namely along the back-azimuth, and this condition would be preserved in isotropic and homogeneous media. Conversely, for anisotropic materials, splitting is observed for all back-azimuths, except when particle motion is originally parallel to either the fast or slow directions, for which splitting is null.

The nearly normal incidence angle of XKS phases at seismic stations yields, net of the Fresnel zone, a good lateral resolution, but not vertical, because, in principle, shear wave splitting may occur anywhere along the travel path above the liquid outer core; nevertheless, several studies point out that the major contribution comes from the lithospheric and asthenospheric mantle LPO, with ɸ aligning parallel to the olivine a-axis, or rather to horizontal flows or extension directions (e.g. Refs.^21,22^). The depth limit of the anisotropic region in the upper mantle is thought to correspond to the Lehmann discontinuity at about 200 km depth (e.g. Refs.^17,23,24^) and references therein), which should mark the transition to isotropic material below. However, in some subduction zones, anisotropy may further extend at least to the olivine-spinel transition at 400 km^17^, with the slab acting as a barrier that forces the mantle to flow parallel to its strike (namely, ɸ parallel to the slab). The assumption of the upper mantle being the major contributor to XKS waves splitting is evidenced by the results from those pairs of phases that sample the upper mantle in a similar way, while the lower differently (as the SKS and SKKS phases generated by the same earthquake); their related splitting parameters match for most cases^17,18^. Nevertheless, albeit with less effect on the final measurements, seismic anisotropy has also been described in the lowermost part of the mantle (i.e. the D’’ layer^25^) and may also be present in the transition zone in some subduction systems^26,27^.

When finally passing through the crust, where layering is horizontal at a large scale, splitting of vertically propagating and horizontally polarised XKS waves is expected to be small^17^; moreover, given the much lower thickness, it is assumed that the parameter dt from the crust is generally one order magnitude smaller than that related to the upper mantle^17,18^. Thus, although for mantle studies crustal anisotropy must be considered as a possible contaminant of the signal, for normal crustal thickness it reasonably only yields a relatively small contribution to the splitting of XKS phases^28^.

In interpreting XKS splitting results one must consider that, given the inertia for mantle minerals to be reoriented, they are not a direct indicator of current stress, which in turn can be investigated by crustal shape-preferred orientation anisotropy.

## Data and results

We investigated the spatial distribution of XKS waves splitting parameters using seismograms recorded by a dense net of 165 broadband stations, belonging to the Italian National Network (http://terremoti.ingv.it/instruments/network/IV) and the Mediterranean Very Broadband Seismographic Network (http://terremoti.ingv.it/instruments/network/MN).

**Figure 2:**
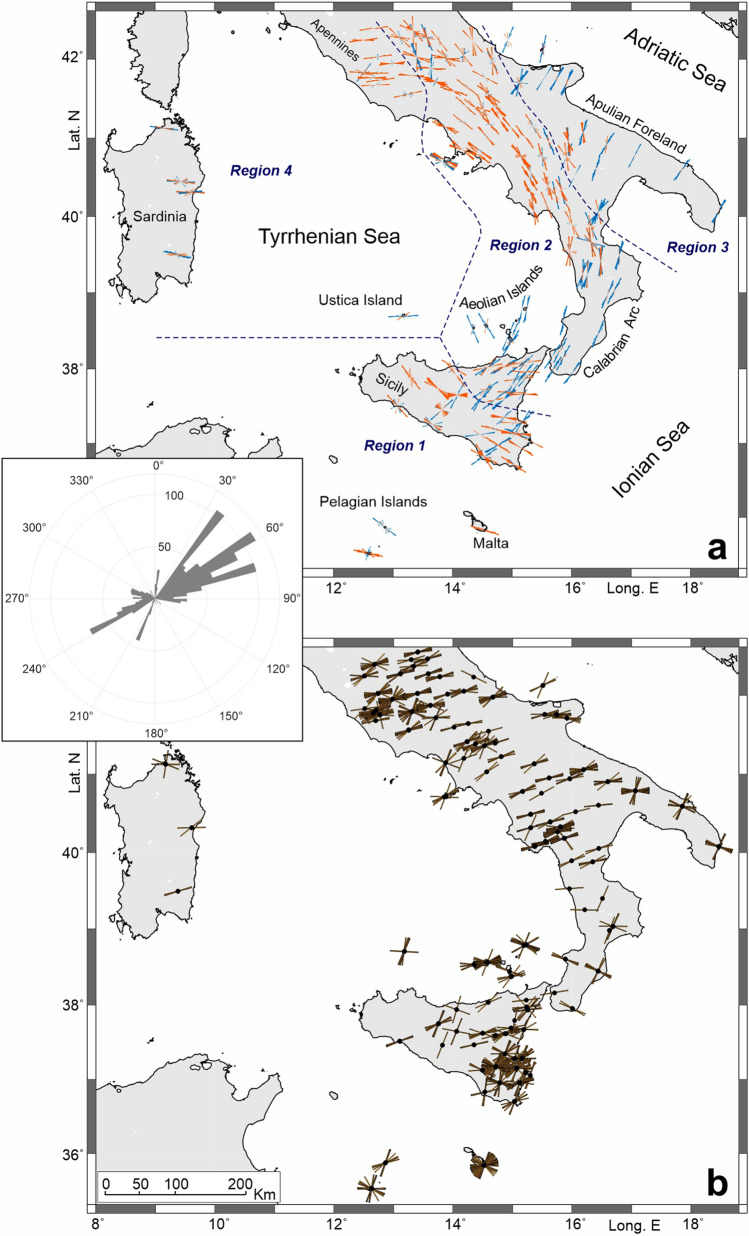
(**a**) Map of the XKS fast directions (ɸ) coloured as a function of the back-azimuths; specifically, ɸ are reported in blue and orange for waves coming from NE and SW, respectively. Four Regions can be distinguished on the base of fast direction trends; for further details see text. (**b**) Back-azimuth of the waves with ‘null’ measurements are shown. The central inset displays back-azimuths of all analysed earthquakes. The maps were created using Generic Mapping Tools (version 6.0; https://www.generic-mapping-tools.org/) and MATLAB (version R2023a; https://it.mathworks.com/products/matlab.html).

Signals from about 1340 earthquakes (central inset in Fig. 1) with a magnitude greater than 6, occurring between 2008 and 2022 at epicentral distances ranging from 85° to 140°, were considered and analysed using the software by Link et al.^29^, which allows full automation in computing ɸ and dt, by using objective criteria. After processing and a qualitative selection (see Methods for details), we obtained 5564 valid splitting measures at 141 stations (Fig. S1 and Table S1 in Supplementary Information); in addition, 1224 ‘null’ results were detected, i.e. S-phases showing no splitting phenomena, which ideally should correspond to events coming from parallel or perpendicular direction to the fast polarisation axis of the anisotropic medium. Measures of fast directions (ɸ) are displayed in Fig. 2a; since earthquakes’ back-azimuths are in two main clusters, namely NE and SW (inset in Fig. 2), for each station we plotted all fast directions with two-coloured symmetrical rose diagrams. Specifically, ɸ are reported in blue and orange for XKS waves coming from NE and SW, respectively. Back-azimuths related to ‘null’ measurements are shown in Fig. 2b.

Rose diagrams show a unimodal distribution of fast directions almost everywhere, although in eastern Sicily a back-azimuthal dependence is evident; in particular, two trends are observed, NE-SW or NW–SE, depending on whether the events come from NE (blue bars in Fig. 2a) or SW (in orange). This pattern likely suggests the presence of prominent lateral variations, essentially because the waves sample different anisotropic mantle regions, according to their back-azimuth (see Fig. S2 in Supplementary Information and Ref.^27^). Other hypotheses related to some structural complexity in the upper mantle, e.g., multiple or dipping anisotropic layers, are not supported by the observations; indeed, no periodic variations of the splitting parameters (diagnostic in such cases) were detected in the back-azimuthal range of the analysed earthquakes dataset.

Overall, based on the fast directions, the central Mediterranean area may be subdivided into four distinct regions (Fig. 2a). ‘Region 1’, in the southern zone, i.e., Malta, the Pelagian Islands and a large part of Sicily, has prevailing NW–SE fast direction, roughly perpendicular to the axis of the Sicilian segment of the Apennine-Maghrebian chain. In ‘Region 2’, which includes central-northeastern Sicily, the Calabrian Arc and the southern Apennines, the azimuth of fast directions is trench-parallel, i.e. NE-SW to the south, gradually rotating to NW–SE northwards. In ‘Region 3’, which consists of the eastern sector of the studied area, i.e., the Apulian foreland, fast directions depict a roughly NE-SW trending vector field, as also found on the opposite shore of the Adriatic Sea (see e.g., Ref.^30^). Finally, ‘Region 4’ comprises the westernmost sector of the area, i.e., Sardinia and the lower Tyrrhenian Sea (see Ustica island in Fig. 2a), and exhibits E-W fast directions; the same polarisation was also found in the Tyrrhenian side of the Apennines, around 42° of latitude. The back-azimuth of the XKS waves with null measurements match with the distribution of the fast or slow directions (Fig. 2b).

**Figure 3:**
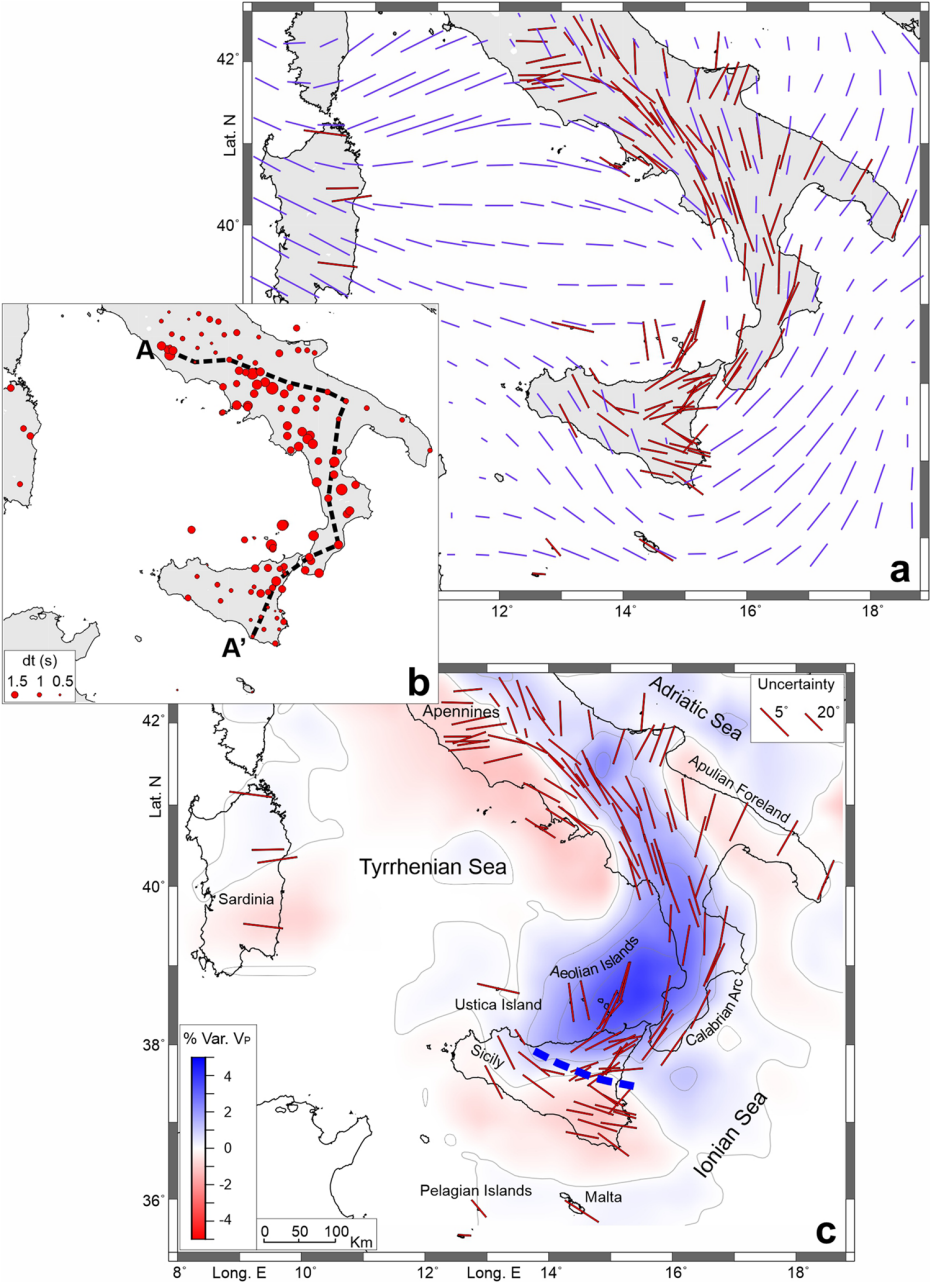
(**a**) Comparison between the XKS waves ɸ-means (in red) and the Pn polarisation (in violet; from Ref.^50^). (**b**) Delay times (dt) as mean for individual stations. The black-dashed line refers to the profile A-A’ in Fig. 4. (**c**) P-wave velocity anomalies at 100 km of depth, derived from Ref.^51^, with the XKS waves ɸ-means superimposed (the same data as reported in **a**). The blue-dashed line identifies a main tectonic lineament (see text for details). The maps were created using Generic Mapping Tools (version 6.0; https://www.generic-mapping-tools.org/) and Surfer (version 21.1.158; https://www.goldensoftware.com/products/surfer).

For those stations showing at least 9 measurements (about 90% of the stations), following Link et al.^29^, we performed a ‘joint splitting analysis’, designed to allow a fast and more objective analysis of a vast amount of data; it works by applying a bootstrap statistics using all phases at a given station to derive an average distribution of the splitting parameters ɸ (ɸ-mean) and dt (dt-mean); results from this analysis are shown in Fig. 3. Standard errors (1σ) are in the order of 10° for ɸ-means and 0.3 s for dt-means. The average fast polarisation values reinforce the overall picture outlined by the single splitting directions. As regards dt-mean values, they vary between 0.37 and 2.8; higher delay times were found in northeastern Sicily, along the Calabrian Arc and the southern Apennines (Fig. 3b).

## Discussion and conclusions

Subduction and collisional zones, such as the Central Mediterranean, are geologically complex, as is their anisotropic pattern; however, by identifying distinct domains with a coherent trend of the fast directions and delay times, a comprehensive geodynamical picture can be outlined.

As discussed earlier, four regions can be distinguished in the study area (Fig. 2a). The anisotropy pattern found within Regions 1, 2 and the northeastern part of Region 4 has generally been attributed to a mantle flow beneath the Adriatic-Ionian subduction system (e.g. Refs.^30–36^). Such flow, triggered by the slab retreat, would develop parallel to the still continuous slab portion, i.e., beneath the southern Apennines and the Calabrian Arc, and would tend to ‘escape’ towards the Tyrrhenian Sea at its edges, namely in Sicily and in the central Apennines. Very similar scenarios, with fast directions trench-parallel in the central area and trench-perpendicular at the slab edges, have been found elsewhere, for instance in the Gibraltar Arc^37^ or in North America^38^.

As regards Region 3, the NE-SW trend of fast directions mirrors the Adria plate motion^39^, which, according to Ref.^40^, has been driven, since the Neogene, by the African plate pushing from the south, and by the Adriatic-Hellenides slab pulling to the northeast. Hence, this direction of anisotropy has been interpreted as a preferred fabric orientation likely deriving from the dragging mechanism that the Adria plate motion generates in the underlying mantle^30^ (although, an active mantle flow would produce a similar trend of fast directions). Then, the E-W vector field found in Region 4 would reflect the upper mantle deformation generated by the Tertiary opening of the Tyrrhenian basin.

Our results mostly fit this general model, but the high spatial density of the sampling points, as well as the great number and quality of the measurements of XKS waves splitting parameters, allowed us to thoroughly investigate the upper mantle setting and discontinuities, in particular in central-eastern Sicily, between the subduction and continental collision domains.

Insights come from the spatial distribution of the delay times between the fast and the slow waves first arrivals (Fig. 3b), as high dt-mean values are observed between northeastern Sicily and the central-southern Apennines, i.e. in Region 2, where fast directions are trench-parallel. There, a sub-slab mantle flow can explain first-order features of the overall anisotropy, while a variety of other mechanisms has been proposed to account for observations in specific zones of the subduction domains. Such models include, for example, trench-parallel anisotropy due to mantle flows above the slab (e.g. Refs.^41,42^) or aligned hydrated faults in the slab itself^43^. Furthermore, it seems that hydrous minerals in the mantle wedge contribute to produce strong trench-parallel seismic anisotropy, which account for the high dt values measured above the slabs (e.g. Refs.^44,45^). Thus, whatever the combination of mechanisms that originate the anisotropy in the subduction zones, a common observation is the marked trench-parallel fast direction and high delay times; such features here represent a distinctive element to delineate the Adriatic-Ionian slab with good accuracy.

**Figure 4:**
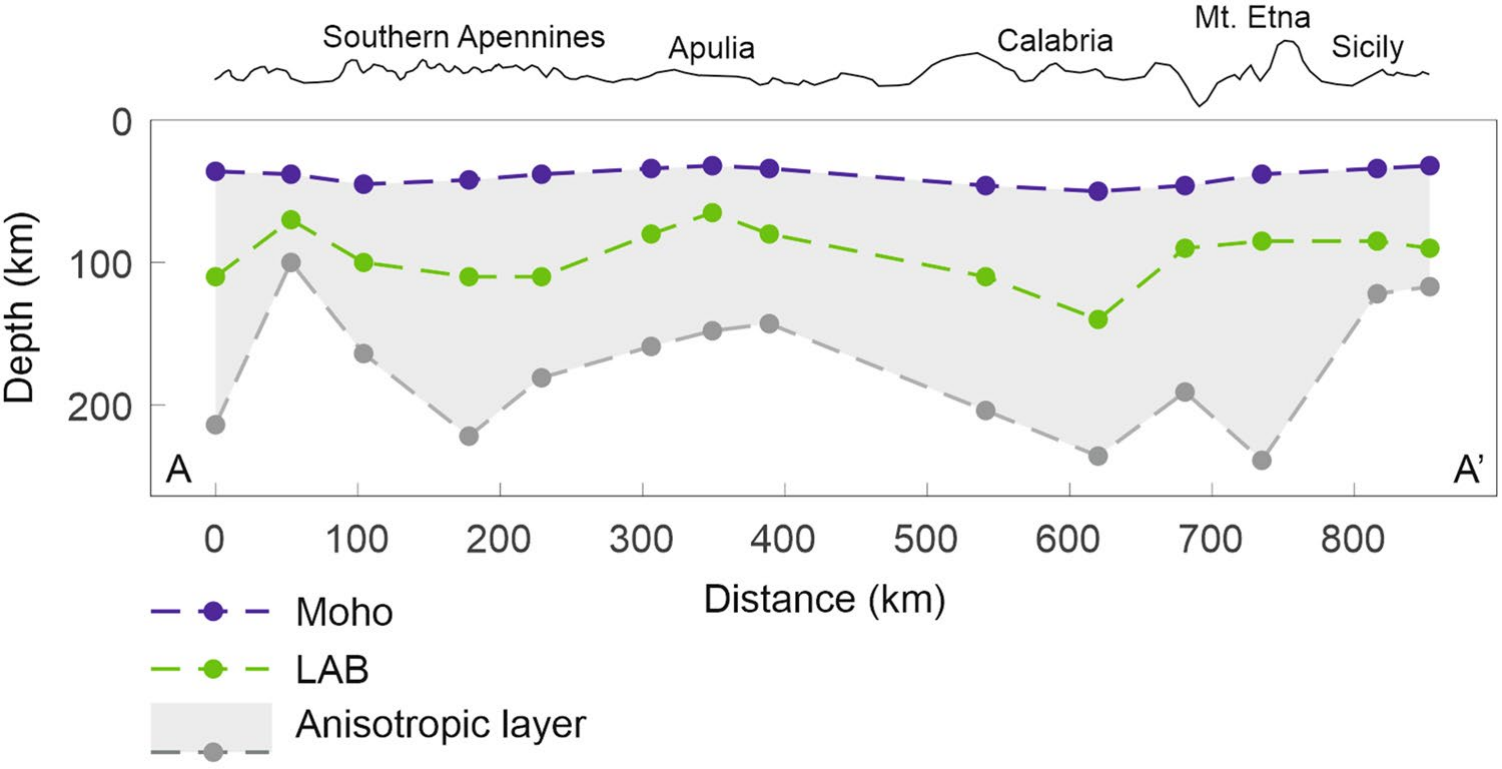
Thickness of the mantle anisotropic layer estimated according to the formula by Refs.^46,47^, along the profile A-A’ traced in Fig. 3b. In the same plot the Moho depth^48^, and the litho-asthenospheric boundary (LAB)^49^ are shown as well.

Regarding the depth extent of the anisotropic layer, it is possible to estimate its thickness beneath seismic stations using the formula by Silver and Chan^46^ and Helffrich^47^, which accounts for the dt-mean values, a shear wave velocity of 4.48 km/s and a 4–5% of anisotropy percentage in the upper mantle. Following this criterion, in Fig. 4 we show a profile running along the whole study area (trace in Fig. 3b), where the depth of the Moho^48^ and of the litho-asthenospheric boundary (LAB)^49^ are shown, together with the estimated thickness of the anisotropic layer. It indicates that the anisotropy in the upper mantle involves, at least partially, the asthenosphere.

To investigate whether the anisotropic asthenosphere and overlying lithosphere deform coherently we compared the polarisation of XKS waves with that of Pn phases, these last related to the head-waves sub-horizontally travelling immediately below the Moho. Anisotropy detected by the Pn phases is representative of the shallow lithospheric mantle^17,18^. In southern Italy, the XKS ɸ-mean and the polarisation of Pn waves, derived from Diaz et al.^50^, show a fairly good congruence (red and violet bars, respectively, in Fig. 3a). A noticeable difference between the two vector fields is only observed in the southern part of the study area where, while the Pn polarisation trend shows a gradual rotation from NE-SW in the Calabrian Arc and the Ionian Sea to NNW-SSE in central-western Sicily, XKS ɸ-mean reveals a sharp variation in fast directions between northeastern and the rest of Sicily.

The comparison with several available seismic tomographies (e.g. Refs.^3,51–53^) further strengthens the overall picture. In Fig. 3c we show a horizontal layer of the P wave velocity anomalies at 100 km of depth, derived from Ref.^51^, with the XKS waves ɸ-mean vector field superimposed (additional tomographic images are in Supplementary Information, Fig. S3). Impressive correspondences can be found; the zone characterised by trench-parallel fast directions and high dt values matches the one where the positive V_P_ anomaly marks the presence of the slab. More in detail, the map shows a roughly WNW trending boundary between northeastern Sicily and the rest of the island (blue line in Fig. 3c); it is straightforward by considering the abrupt change in both XKS phases’ splitting parameters (ɸ-means and dt-means, Fig. 3) and seismic tomography, pointing to the existence of a sharp mantle discontinuity.

It is worth noting that, right along the above-mentioned WNW trending boundary, Barreca et al.^4^, based on geological, gravimetric and seismological data, identify an important structural alignment, described as a deep-seated shear zone. It would have played the role of a tearing zone (Subduction Transform Edge Propagator fault, STEP in the sense of Govers and Wortel^54^), which allowed the rapid trench migration produced by the south-eastward rollback of the Ionian slab in northeastern Sicily and the Calabrian Arc^1^, likely also controlling the emplacement of the Etna volcano^10^.

Since there is a precise correspondence of this shear zone observed at the crustal level also in the upper mantle fabric, as can be deduced by the trend of the XKS waves splitting parameters and seismic tomography (Fig. 3), it is reasonable to assume that this discontinuity represents a segmentation or a paleo margin of the subduction system (see e.g. Ref.^55^). It stands in a transitional zone between the continental and the oceanic domains^56^; this setting, along with the rapid Ionian slab rollback and its subsequent near-cessation^16,57^, could explain the rather abrupt switch of the XKS waves fast directions. In this framework, the conditions to generate a proper toroidal flow around the slab would be lacking; thus, the picture we observe would rather represent the juxtaposition of the lattice-preferred orientation in two contiguous sectors of the mantle: one fast direction, trench-parallel, i.e. NE-SW in northeastern Sicily, is related to the existence of the slab, and the other, i.e. NW–SE in southern and central-western Sicily, is the one taken by the mantle in relation to the relative Nubia and Eurasian plates motion (e.g. Ref.^58^). This setting would also be supported by the back-azimuthal dependence of fast directions found in this area, where XKS waves sample the foreland upper mantle when coming from the southwest (shown as orange bars in Fig. 2a), and the Ionian slab related mantle when coming from the northeast (shown in blue in Fig. 2a; see also Fig. S2 in Supplementary Information).

The NNW-SSE fast directions found at Alicudi and Filicudi, the westernmost islands of the Aeolian archipelago (Fig. 2a), could appear anomalous if compared with those of the neighbouring sectors; actually, it further testifies the complex structure and dynamics created at the subduction margin, which led to the segmentation and the opening of slab windows^3^. Indeed, as previously mentioned, west of ATLFS (Fig. 1) the slab consists of a remnant completely detached at shallow levels^3^; through the resulting slab gateway it is possible to hypothesise the occurrence of a mantle flow between the Tyrrhenian fore-arc region and mainland Sicily^15,59^, which could account for the fast directions observed at Alicudi and Filicudi and the magmatic source mixing at Etna volcano (see Ref.^60^ and reference therein).

Moving north of Calabria, the high velocity anomalies characterising the slab in seismic tomography (Fig. 3b) are less sharp if compared to those of the southern sector, where the slab is considered to be continuous at depth (see also Fig. S3 in Supplementary Information). Scarfì et al.^3^ highlight the presence of a horizontal breakoff that would interrupt the continuity of the slab, roughly in northern Calabria. There, XKS fast directions were found as trending mostly parallel to the trench, but some measurements indicate an almost perpendicular direction (see Fig. 2a), perhaps related to an incipient window in the slab, through which currently no significant mantle flow may occur. Conversely, a prominent mantle flow towards the Tyrrhenian Sea, due to a slab window or tear, was hypothesised in the central Apennines^30,36^ (northeastern Region 4, around 42° of latitude; Fig. 2a); this picture is in agreement with our findings, which evidence a rotation from NW–SE to E-W of fast directions, and is also supported by the marked discontinuity in the V_P_ anomalies (Fig. S3).

**Figure 5:**
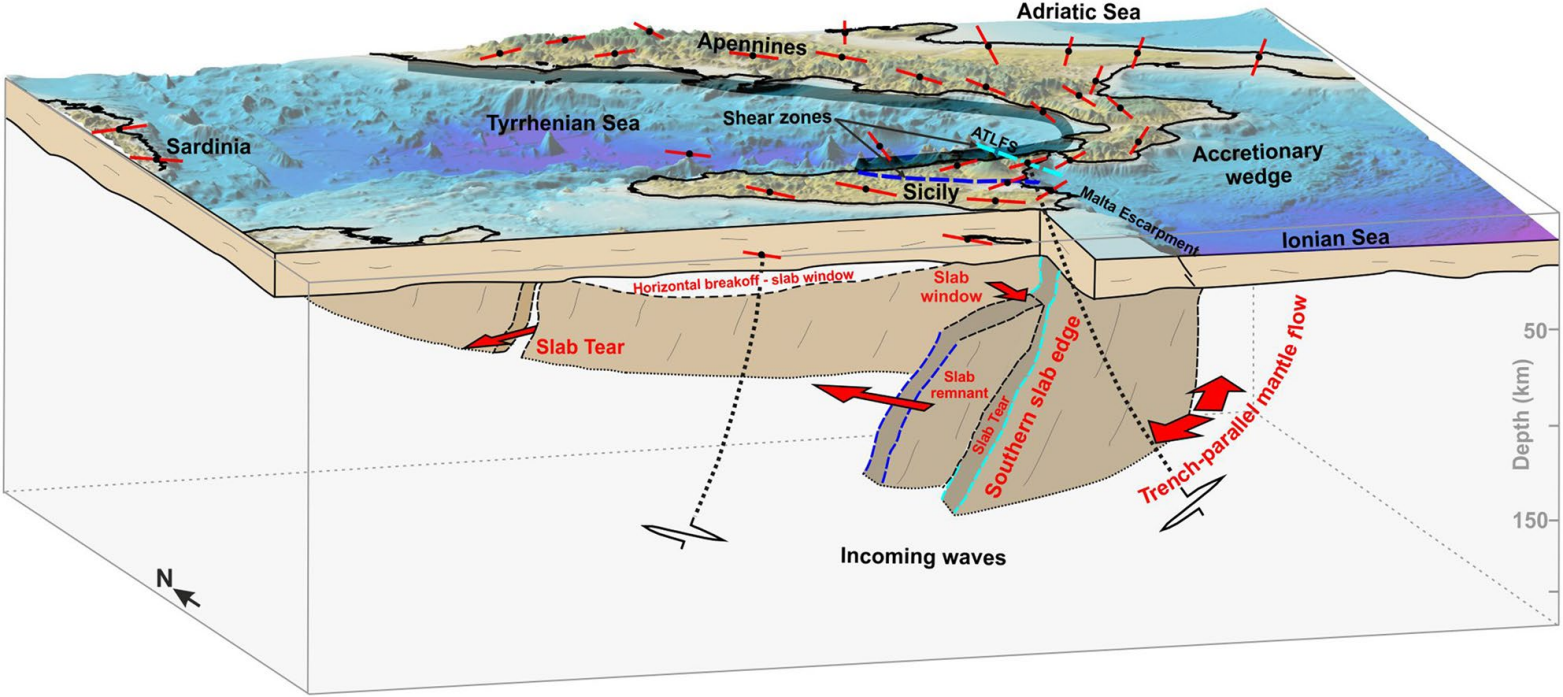
Schematic conceptual model illustrating the mantle flow around the subduction system, interpreted according to the results from this study. The orientation of red bars indicates the average fast polarisation direction (ɸ-mean) at selected stations. The thick grey line along the Tyrrhenian coast indicates the slab at depth. The dashed cyan line (at the surface and depth) indicates the STEP fault bounding the still active slab portion; the dashed blue line would represent a paleo-margin or paleo-STEP of the subduction system. Red arrows indicate the inferred directions of mantle flow. Topography is from Ref.^67^. The map was created using Surfer (version 21.1.158; https://www.goldensoftware.com/products/surfer); the figure was edited using CorelDraw Graphics Suite (version 2020; http://www.corel.com).

In conclusion, the high resolution of XKS waves splitting observations in southern Italy highlights details which contribute to better constraining the structural architecture of the Ionian subduction zone, also linking shallow tectonic features with the deeper ones (Fig. 5). Primarily, other than accurately outlining the slab, our results highlight a sharp discontinuity in the upper mantle fabric in the southern study area. This deformation propagates through the whole lithosphere, slicing the entire central-eastern Sicily through a WNW structural belt^4,10^; it may effectively represent the main lineament of a STEP fault system emplaced in the transitional domain of the collisional margin^56^, while the still active portion of the sinking, oceanic-type Ionian slab is confined more to the northeast, along the ATLFS (see Refs.^3,61^). An upper mantle flow, perhaps developed as a result of detachments and lateral tearing of the subducting lithosphere, accompanied by strike-slip tectonics in the upper plate, may have favoured and controlled tertiary volcanism in eastern Sicily (see e.g. Refs.^4,10,60,62^). The rapid variations between trench-parallel and trench-perpendicular fast directions observed just at the southern edge of subduction seem to suggest the absence of a toroidal flow as previously assumed^32–35^.

It turns out that the study of seismic anisotropy may be fundamental to investigate the continuation of major tectonic structures at depth, down to the upper mantle, and could represent a mandatory step for a proper understanding of active subduction systems.

## Methods

Seismograms were analysed by using an automatized software package developed by Link et al.^29^; this is an extension of the ‘SplitRacer’ MATLAB toolbox^63^ and has been designed for full automation of the computing of splitting parameters. The software allows the user to download data from different sources using the FDSN Web Services (International Federation of Digital Seismograph Networks).

Since core-mantle phases actually produce a marked change in the spectral content on seismograms, the software is capable of automatically identifying XKS waves by performing a time-dependent spectral analysis on a window centred around the expected arrival times, derived using the IASP91 velocity model^64^. After applying a band-pass filter (which we set to 4–20 s to attenuate the energy of noise-related harmonic components) and using a threshold based on STA/LTA signal-to-noise ratio, a quality check is automatically performed on the seismic traces, using objective criteria (see Ref.^29^ for details).

Noise-dominated or uncertain traces are then discarded, while the remaining ones are analysed to estimate the splitting parameters ɸ, i.e. the azimuth of polarisation of fast wave, and dt, i.e. the delay time between the fast and the slow waves first arrivals, through the transverse component minimization method by Silver and Chan^21^. This methodology is meant to solve splitting parameters for pulse duration longer than dt, as in the case of XKS waves; in principle, in the absence of anisotropy, XKS waves particle motion is entirely along the radial component (i.e. along the back-azimuth), while, after crossing an anisotropic medium, splitting occurs and significant energy is also found on the transverse component, producing elliptical particle motion. Therefore, for pulse duration shorter than dt, a straightforward method to derive the fast (ɸ) and slow polarisation azimuths is to rotate the reference axes of horizontal seismograms until the first shear arrival is seen entirely on the fast component, separated from the second shear arrival on the slow component. Instead, for pulse duration longer than dt, the fast and slow components will not achieve a full separation in time on the seismograms and therefore interference will take place for all azimuths. Silver and Chan^21^ demonstrated that in the latter condition, the transverse component is proportional to the time-derivative of the radial one. In principle, as no splitting occurs whenever the back azimuth is parallel to either the fast or the slow directions, with all energy concentrated in the radial component, splitting parameters can be analytically found by searching for such a condition. Therefore, horizontal seismograms are firstly rotated into the radial and transverse directions and then those azimuths and dt that minimise the energy of the transverse component yield the splitting parameters, ɸ being that azimuth showing the first shear arrival (see Fig. S4 in Supplementary Information).

In addition, the software by Link et al.^29^ includes the calculation of the splitting intensity^65^ and the rotation correlation^66^, for comparing results with those formerly calculated following Silver and Chan^21^, allowing the formulation of thresholds to objectively classify the measurements into: i) ‘good’ and ‘average’, with clear split of the waves, a linearized particle motion after correction and a small area of 95% confidence interval of uncertainty; ii) ‘poor’, with insufficient or no possible linearization after correction and a relatively large area of 95% confidence level. Finally, the measurements are classified as ‘null’ when no anisotropy is detected (ideally, when the initial particle motion is parallel or perpendicular to the fast polarisation axis of the anisotropic medium; see Refs.^29,63^ for further details). Results are shown in Fig. 2.

As a further step, we performed a ‘joint splitting analysis’ by applying bootstrap statistics considering all measurements, including ‘nulls’, at a given station in order to derive an average distribution of the splitting parameters (see Ref.^29^ for further details); results are shown in Fig. 3.

### Supplementary Information

Supplementary Information.

## Data Availability

The earthquake parameters (locations and origin times) are from the USGS earthquake catalog (https://earthquake.usgs.gov/earthquakes/search/). Analysed waveforms are from INGV FDSNWS DataSelect Web Service (http://webservices.ingv.it/fdsnws/dataselect/1/). The software used for waveform analysis is by Link et al. [2022; available at https://www.geophysik.uni-frankfurt.de/64002762/Software].
