# Supplementary material for: Seismic anisotropy to investigate lithospheric-scale tectonic structures and mantle dynamics in southern Italy
Source: Sci Rep. 2023 Nov 28;13:20932. doi: 10.1038/s41598-023-47973-1 (PMC10684859; doi:10.1038/s41598-023-47973-1)
Supplement: Supplementary file 1 — Supplementary Information. [file 41598_2023_47973_MOESM1_ESM.pdf]

Seismic anisotropy to investigate lithospheric-scale tectonic structures and mantle dynamics in southern Italy

Scarfì L.*, Firetto Carlino M., Musumeci C.

Istituto Nazionale di Geofisica e Vulcanologia – Osservatorio Etneo, Catania, Italy

Corresponding author: luciano.scarfi@ingv.it

Introduction

This supplementary material provides information about the results of the splitting analysis at 141 seismic stations.

Seismic network

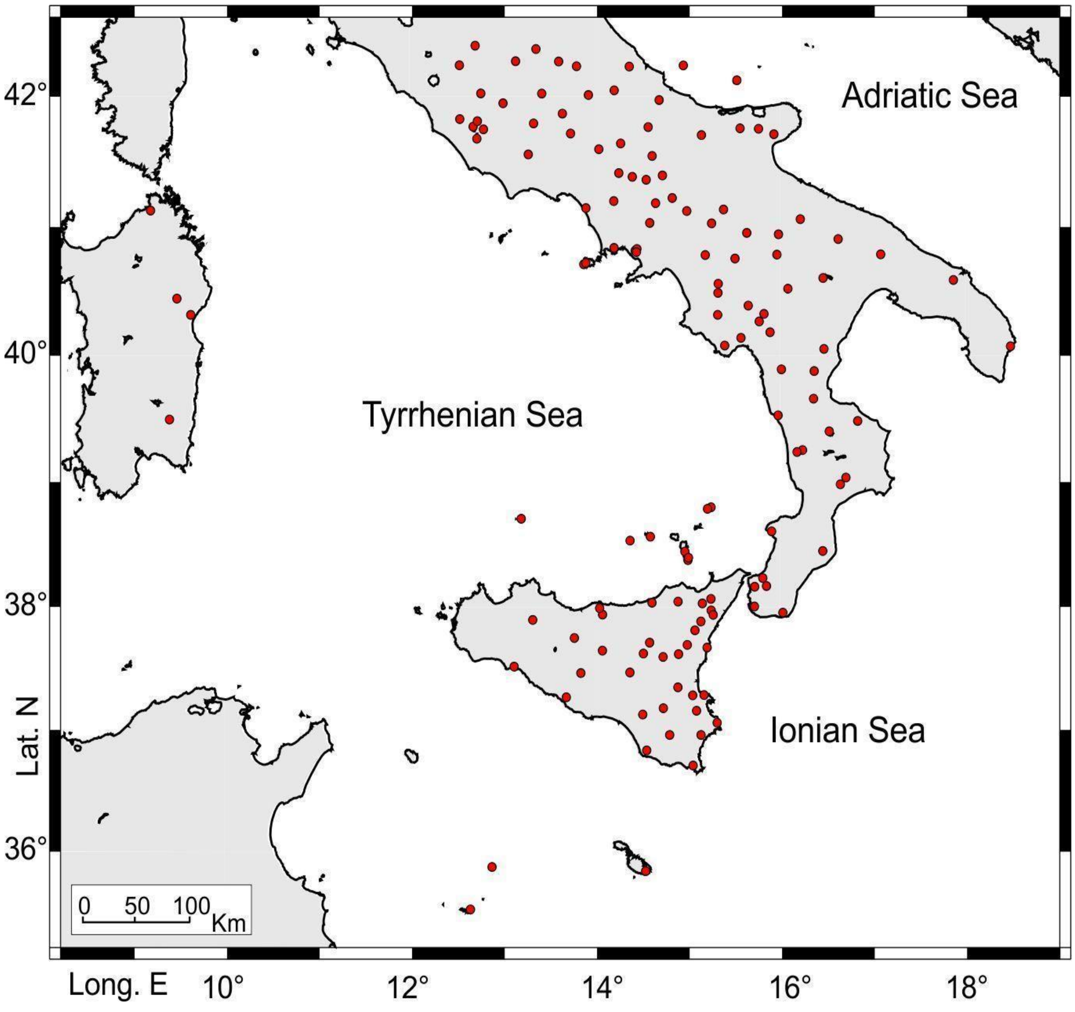

Figure S1. The red dots indicate the seismic stations used in the study. The map was created using the software Generic Mapping Tools (version 6.0; https://www.generic-mapping-tools.org/).

Results

Table 1. It shows the position of the analyzed stations, the number of splitting measurements classified as null, good and average, and the ϕ- and dt-mean values and the relative uncertainties obtained through the bootstrap procedure (see Methods in the main text for details). For stations with less than 9 useful measurements, the bootstrap value is not indicated; in this case, the fast directions are shown in Fig. 2.

Station code	Lat. (N)	Long. (E)	Null	Good	Average	ϕ-mean (°)	Uncert. (°)	dt-mean (s)	Uncert. (s)
ACER	40.787	15.943	-	6	8	-14.33	23.68	1.25	0.26
AGLI	41.127	9.173	5	33	20	-82.60	1.72	1.54	0.11
AIO	37.971	15.233	5	19	42	57.93	14.57	0.85	0.13
ALJA	37.749	13.754	13	9	20	-51.97	5.20	1.13	0.21
AMUR	40.907	16.604	7	48	21	15.28	6.79	1.17	0.14
APRC	41.757	15.543	3	23	7	28.79	4.65	1.29	0.15
BSSO	41.546	14.594	1	3	24	-23.48	23.30	1.11	0.67
BULG	40.078	15.378	15	15	32	-15.61	1.96	1.52	0.09
CAFE	41.028	15.237	-	8	17	-40.52	2.63	1.81	0.13
CAFR	42.227	14.347	1	1	2	-	-	-	-
CAGR	37.622	14.500	5	5	9	56.67	17.93	1.15	0.56
CARO	39.254	16.219	-	2	3	-	-	-	-
CAR1	39.253	16.211	1	5	23	9.64	3.86	1.65	0.18
CDRU	40.490	15.305	18	33	61	-23.78	1.70	1.86	0.10
CELI	39.403	16.509	1	22	52	1.09	1.37	2.39	0.10
CERA	41.598	14.018	2	10	35	-46.91	7.14	1.20	0.18
CERT	41.949	12.982	34	18	28	81.52	3.54	1.08	0.30
CET2	39.529	15.955	1	4	6	2.49	6.97	2.01	0.49
CMDO	37.464	13.823	1	-	11	-53.81	5.81	1.11	0.18
CMPR	40.318	15.303	8	29	59	-28.92	2.32	1.69	0.12
CMSN	40.838	14.182	-	2	6	-	-	-	-
CORL	37.894	13.304	-	5	19	-26.38	7.00	1.00	0.17
CROCE	36.833	14.535	5	4	14	-78.87	11.12	0.86	0.15
CSLB	37.937	14.058	2	-	4	-	-	-	-
DGI	40.318	9.607	2	24	29	81.65	4.61	1.49	0.26
ECNV	37.596	14.713	1	8	8	75.26	6.97	1.73	0.56
ECTS	37.882	15.121	-	1	1	-	-	-	-
EMCN	37.791	15.034	1	7	4	28.36	12.19	1.33	0.66
EPIT	37.811	15.057	-	11	5	37.10	3.40	2.02	0.19
EPOZ	37.672	15.189	4	9	25	84.97	4.11	1.62	0.40
ESLN	37.693	14.974	2	4	21	87.75	22.62	1.38	0.29

ESML	37.618	14.879	12	14	10	53.53	4.78	1.27	0.36
FAGN	42.266	13.584	12	23	41	-18.19	2.97	1.36	0.16
FAVR	37.267	13.667	-	2	4	-	-	-	-
FIAM	42.268	13.117	27	18	31	-36.51	27.32	0.57	0.25
FRES	41.974	14.669	18	7	14	-8.75	14.16	0.93	1.11
FX01	37.059	15.296	2	3	1	-	-	-	-
GALF	37.711	14.567	-	5	22	70.66	14.97	0.92	0.47
GIB	37.990	14.026	-	1	3	-	-	-	-
GIUL	41.558	13.255	20	1	10	76.66	5.21	0.62	0.22
GMB	38.168	15.829	-	34	35	31.60	3.69	1.72	0.12
GUAR	41.795	13.312	36	14	16	-77.26	6.29	0.85	0.25
HAGA	37.285	15.155	20	2	36	41.08	6.68	0.68	0.28
HAVL	36.960	15.122	35	22	8	-81.29	8.06	0.76	0.15
HBSP	37.127	14.492	4	4	4	-78.97	17.10	0.75	0.25
HCRL	37.283	15.033	13	5	20	-61.12	19.76	0.43	0.23
HLNI	37.349	14.872	20	25	35	-54.45	7.76	0.76	0.15
HMDC	36.959	14.783	20	62	51	-72.63	3.64	0.99	0.10
HPAC	36.709	15.037	11	9	20	-53.18	6.27	1.20	0.27
HSRS	37.093	15.222	5	4	1	-73.27	20.55	1.32	0.28
HVZN	37.178	14.716	61	50	53	-71.05	2.87	0.89	0.11
IACL	38.533	14.355	7	3	6	-7.19	5.50	1.37	0.27
IFIL	38.564	14.575	42	7	16	-12.18	4.46	0.63	0.30
IFOR	40.712	13.855	2	4	7	-56.79	8.84	1.22	0.28
ILOS	38.446	14.948	2	3	6	25.45	2.30	2.29	0-34
IMTC	40.721	13.876	5	2	3	-79.66	43.20	1.14	1.84
INTR	42.012	13.905	3	28	38	-37.26	9.14	1.06	0.20
IST3	38.799	15.230	12	17	21	13.92	3.86	1.97	0.34
ISTR	38.787	15.192	11	16	28	16.44	2.10	2.28	0.26
IVPL	38.376	14.981	4	9	28	41.90	5.90	1.47	0.21
IVUG	38.396	14.986	-	8	1	25.87	5.89	1.46	0.17
JOPP	38.607	15.886	3	28	31	10.80	1.46	2.18	0.11
LAV9	41.678	12.699	2	3	14	84.49	3.29	2.28	0.78
LINA	35.872	12.863	10	2	4	-39.92	21.41	0.37	0.12
LPDG	35.518	12.630	15	3	22	-86.08	40.38	0.41	0.30
LPEL	42.047	14.183	6	3	17	-2.18	9.15	1.40	0.23
MA9	41.770	12.659	12	15	17	89.27	2.42	1.79	0.27
MCEL	40.325	15.802	27	31	57	-16.90	1.54	2.07	0.13
MCRV	40.783	15.168	9	27	40	-25.35	1.92	1.40	0.14
MCSR	38.065	15.230	1	3	6	80.05	10.71	1.49	0.41

MESG	40.589	17.850	15	74	37	30.00	4.24	1.06	0.10
MELA	41.706	15.127	-	10	31	17.95	7.89	1.58	0.26
MGR	40.138	15.554	20	19	29	-18.99	1.61	1.97	0.12
MIDA	41.642	14.254	2	34	31	-51.51	3.79	1.22	0.11
MIGL	40.604	16.441	2	67	27	14.30	2.58	1.10	0.08
MMME	37.935	15.254	8	4	14	58.82	23.86	0.70	0.36
MMN	39.891	15.990	2	1	8	-20.28	5.91	1.53	0.52
MNS	42.385	12.681	13	3	5	84.87	15.81	0.65	0.74
MODR	41.146	13.878	9	24	30	-61.64	3.24	1.52	0.13
MPAZ	37.953	16.007	2	45	11	35.41	1.92	1.96	0.10
MRB1	41.123	14.968	-	8	30	-37.46	2.01	2.63	0.18
MRCB	38.162	15.703	1	4	2	-	-	-	-
MRLC	40.756	15.489	1	3	11	-14.39	8.08	1.67	0.30
MRVN	41.061	16.196	23	64	65	-7.28	2.76	1.26	0.14
MSAG	41.712	15.910	6	76	58	19.93	1.55	1.10	0.08
MSCL	38.232	15.790	-	22	16	35.87	4.59	1.70	0.15
MSFR	38.034	14.592	2	2	21	55.53	7.53	1.68	0.53
MTCE	42.023	12.742	28	27	29	77.47	4.38	1.09	0.56
MTSN	40.266	15.752	38	39	47	-15.80	1.29	2.12	0.12
MTTG	38.003	15.700	-	47	38	37.04	3.92	1.73	0.14
MUCR	38.043	14.874	-	9	24	62.52	4.24	1.65	0.19
NOCI	40.789	17.064	24	78	45	24.90	2.30	1.14	0.10
NOV	38.028	15.137	-	22	32	64.40	9.01	1.23	0.21
ORI	40.051	16.450	2	20	22	19.42	9.15	1.10	0.10
PALZ	40.944	15.960	6	26	28	-15.50	3.09	1.49	0.11
PAOL	41.031	14.567	2	63	33	-38.25	1.52	1.71	0.11
PIGN	41.200	14.180	2	51	40	-58.46	6.46	1.45	0.15
PIPA	39.485	16.816	-	29	38	10.44	2.31	1.71	0.08
PLAC	38.449	16.438	10	59	66	26.63	1.46	1.87	0.10
POFI	41.717	13.712	6	31	28	-64.33	10.13	0.84	0.16
PSB1	41.223	14.811	3	5	11	-29.46	3.37	1.99	0.24
PTQR	42.022	13.401	20	1	15	-19.53	16.50	0.95	0.43
PTRJ	41.364	14.529	18	-	19	-29.93	18.36	2.43	0.81
PTRP	40.521	16.061	1	-	1	-	-	-	-
RESU	37.647	14.057	3	-	18	-77.03	20.42	0.90	0.41
RMP	41.812	12.705	21	34	29	70.52	9.63	0.88	0.34
ROM9	41.828	12.516	2	17	15	89.47	16.98	1.90	0.73
ROSPO	42.236	14.932	-	4	2	-	-	-	-
SACR	41.397	14.706	3	7	20	-25.09	13.98	1.81	0.51

SALB	39.877	16.346	4	4	12	-16.26	4.13	2.17	0.35
SCIAC	37.516	13.102	6	1	7	-27.46	7.00	1.36	0.75
SCTE	40.072	18.468	16	59	41	22.38	2.39	1.04	0.11
SELL	38.983	16.628	1	7	5	22.12	2.78	1.76	0.11
SENA	40.444	9.457	-	3	7	88.87	11.96	1.33	0.31
SERS	39.036	16.689	7	66	59	24.95	1.71	1.84	0.10
SGG	41.387	14.379	10	27	36	-37.18	6.83	1.50	0.20
SGO	40.560	15.307	-	2	5	-	-	-	-
SGRT	41.755	15.744	7	72	53	20.54	3.15	1.17	0.10
SGTA	41.135	15.365	5	-	12	-24.42	6.42	1.44	0.40
SIRI	40.182	15.868	32	38	44	-17.29	1.66	2.06	0.15
SLCN	40.390	15.633	9	4	14	-17.05	3.37	1.94	0.20
SOLUN	38.092	13.533	24	-	12	-32.59	25.73	0.39	0.23
SRES	42.237	12.510	7	5	8	-88.42	12.66	0.93	0.61
SSY	37.158	15.074	22	29	71	-76.11	2.55	0.92	0.09
TDS	39.660	16.338	-	23	33	-18.66	14.80	1.76	0.58
TREM	42.123	15.510	3	18	18	4.96	6.39	1.41	0.15
TRIV	41.767	14.550	-	1	5	-	-	-	-
T0104	42.360	13.338	3	7	7	-31.82	9.38	1.04	0.15
T0110	42.229	13.776	6	15	23	-28.45	6.00	1.25	0.30
USI	38.708	13.179	19	8	27	-77.00	1.50	1.63	0.22
VAE	37.469	14.353	2	-	2	-	-	-	-
VAGA	41.415	14.234	10	44	25	-43.98	1.10	1.71	0.08
VARP	40.817	14.410	-	3	3	-	-	-	-
VBKN	40.830	14.430	-	17	22	-54.72	2.40	2.00	0.18
VITU	41.183	14.630	-	20	35	-37.51	3.92	2.08	0.24
VIVA	41.750	12.770	8	7	10	80.69	12.11	1.80	1.07
VSL	39.496	9.378	2	28	24	-83.07	4.62	1.18	0.21
VTIR	40.806	14.424	-	9	18	-47.90	5.77	1.73	0.43
VULT	40.955	15.616	8	2	17	-15.79	2.20	1.49	0.14
VVLD	41.870	13.623	11	4	17	-11.90	34.33	0.62	0.32
WDD	35.837	14.524	80	13	23	-57.03	4.53	0.42	0.09

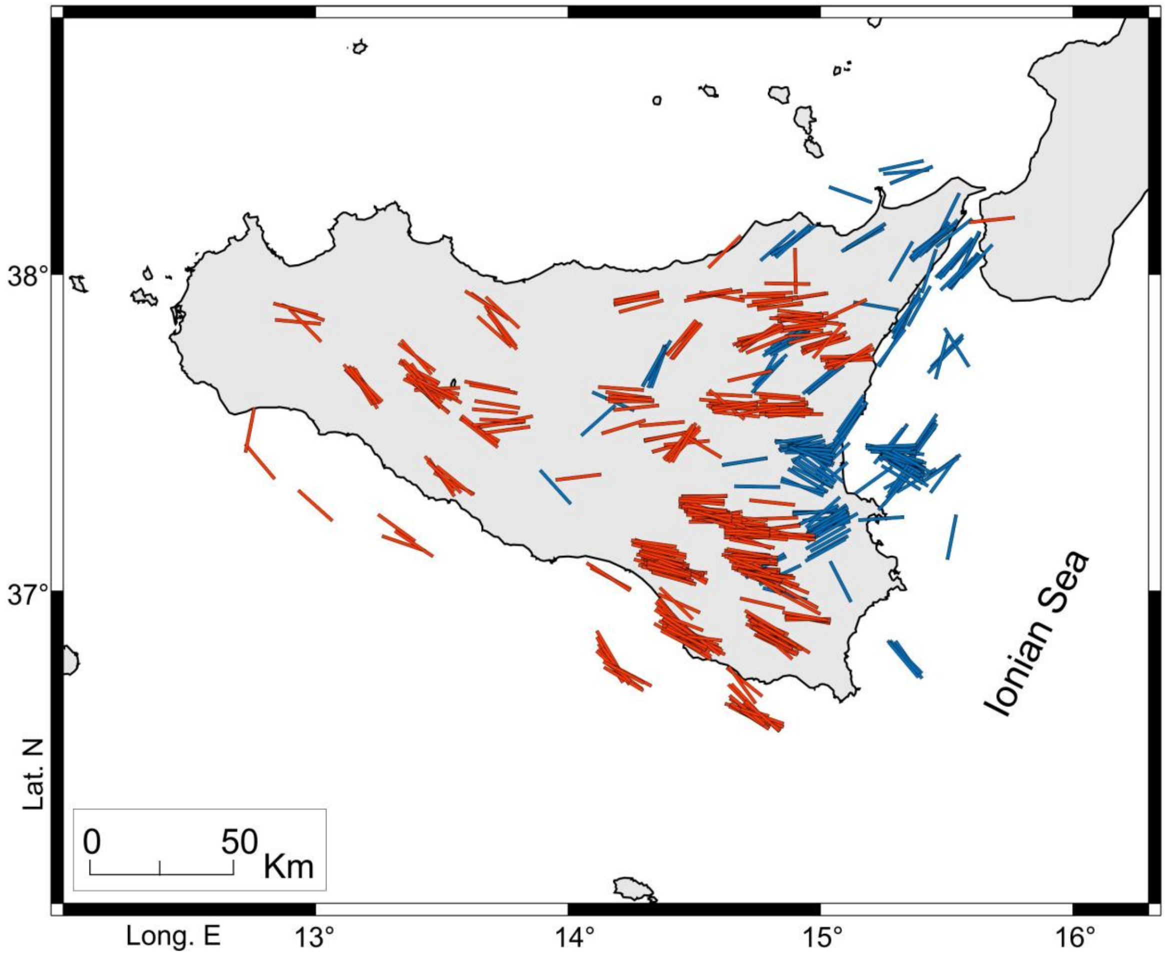

Figure S2. SKS splitting measurements in Sicily, plotted at the 150 km depth piercing points. They are coloured as a function of the back-azimuths; specifically, ϕ are reported in blue and red for XKS waves coming from NE and SW, respectively. The map was created using the software TauP Toolkit (Crotwell et al., 1999; http://www.seis.sc.edu/TauP) and Generic Mapping Tools (version 6.0; https://www.generic-mapping-tools.org/).

Seismic Tomography

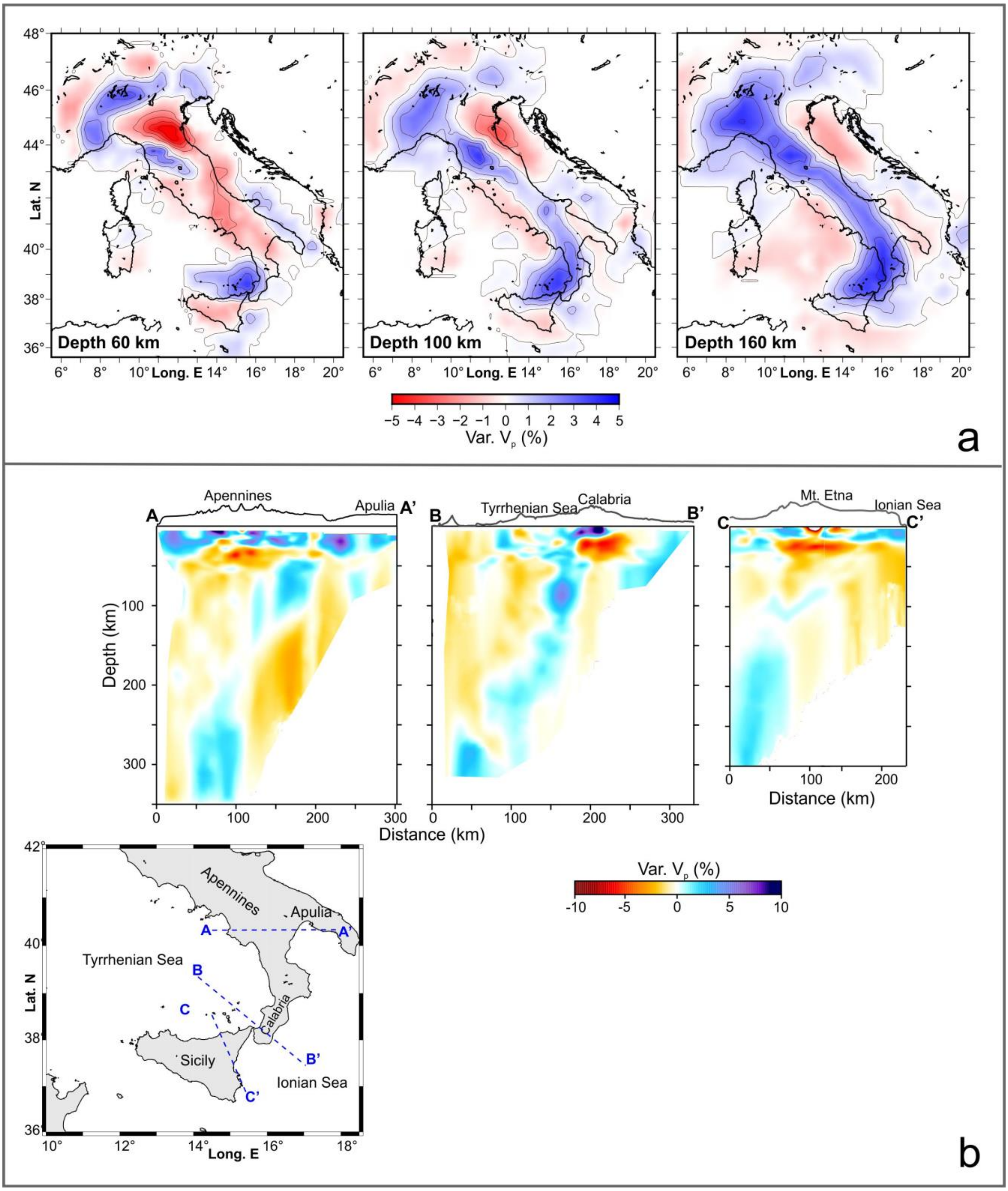

Figure S3. a) V_P anomalies, as perturbations (%) of the initial velocity model, at three depth levels, by Giacomuzzi et al. (2011). b) Vertical sections through the V_P model by Scarfì et al. (2018). The traces of the sections (AA', BB', CC') are reported in the sketch map in the bottom left. The maps were created using the software Generic Mapping Tools (version 6.0; https://www.generic-mapping-tools.org/) and Surfer (version 21.1.158; https://www.goldensoftware.com/products/surfer).

Splitting Analysis

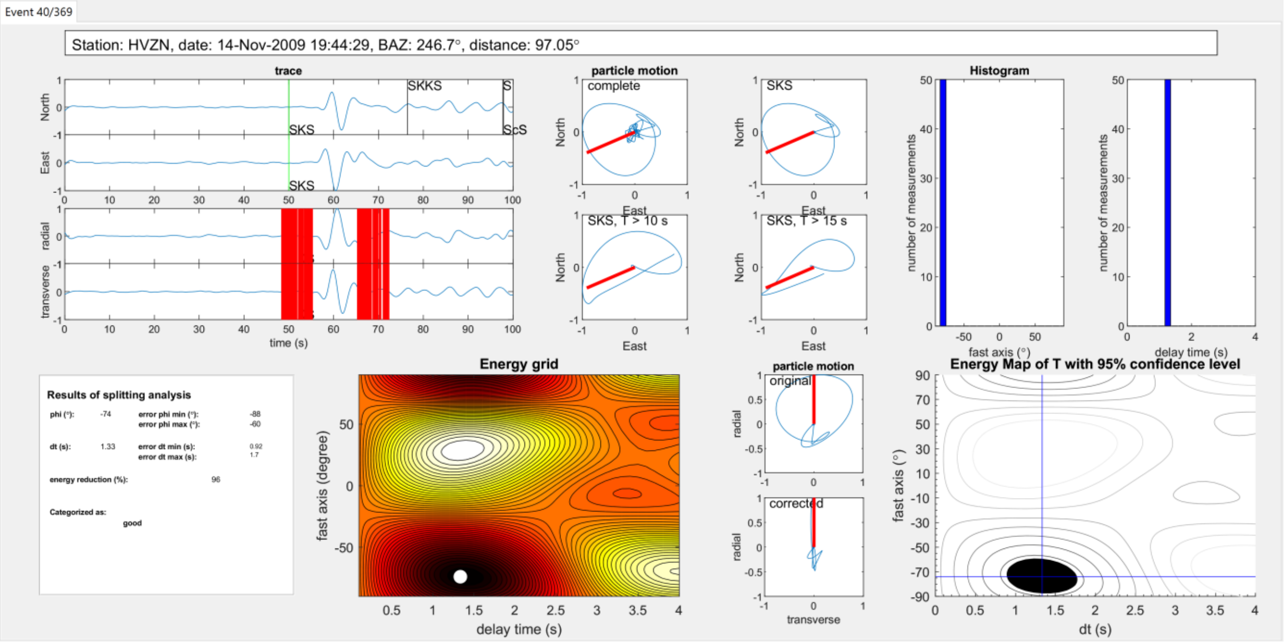

Figure S4. Graphical user interface for Single-phase Splitting Analysis of the ‘SplitRacer’ software (Reiss and Rumpker, 2017) showing an event recorded on November 14, 2009 at station HVZN (Sicily). In the top panel to the left, a 100s zoom into the phase is shown in NE and RT components. In the radial/transverse plot, vertical red lines show the 50 different time windows used for the analysis. In the top centre, particle motions are shown (clockwise rotation from top-left): the complete trace, SKS window, SKS window filtered with a bandpass of 10-50s and 15-50s, respectively. The top right histograms show the distribution of splitting parameters over the 50 time windows. In the bottom left panel, the information box states the result of the splitting analysis, error bars and energy reduction. Following, it is shown: a mean energy grid (white dot marks the splitting parameters); the original and corrected particle motions of RT components; the 95% confidence level (blue cross marks the final splitting parameters).

References

Crotwell, H.P., Owens, T.J. & Ritsema, J. The TauP Toolkit: Flexible seismic travel-time and ray-path utilities. *Seismol. Res. Lett*. **70**, 154-160, https://doi.org/10.1785/gssrl.70.2.154 (1999).

Giacomuzzi, G., Chiarabba, C., & De Gori, P. Linking the Alps and Apennines subduction systems: New constraints revealed by high-resolution teleseismic tomography. *Earth Planet. Sci. Lett.* **301**(3–4), 531–543. https://doi.org/10.1016/j.epsl.2010.11.033 (2011).

Reiss, M.C. & Rümpker, G. SplitRacer: MATLAB Code and GUI for semiautomated analysis and Interpretation of teleseismic shear-wave splitting. *Seismol. Res. Lett.* **88** (2A), 392-409. https://doi.org/10.1785/0220160191 (2017).

Scarfì, L. et al. Slab narrowing in the central Mediterranean: The Calabro-Iionian subduction zone as imaged by high resolution seismic tomography. *Scientific Reports* **8**, 5178. https://doi.org/10.1038/s41598-018-23543-8 (2018).
